# ﻿*Sinoseneciopingwuensis* (Asteraceae, Senecioneae), a new species from northern Sichuan, China

**DOI:** 10.3897/phytokeys.218.97485

**Published:** 2023-01-12

**Authors:** Xiu-Jiang Su, Wen-Qun Fei, Ding Zhao, Ying Liu, Qin-Er Yang

**Affiliations:** 1 Key Laboratory of Plant Resources Conservation and Sustainable Utilization, South China Botanical Garden, Chinese Academy of Sciences, Guangzhou 510655, Guangdong, China South China Botanical Garden, Chinese Academy of Sciences Guangzhou China; 2 Administration Bureau of Baiyunshan Nature Reserve, Baojing 416500, Hunan, China Administration Bureau of Baiyunshan Nature Reserve Baojing China; 3 University of Chinese Academy of Sciences, Beijing 100049, China University of Chinese Academy of Sciences Beijing China; 4 Administration Bureau of Xuebaoding National Nature Reserve, Pingwu 622550, Sichuan, China Administration Bureau of Xuebaoding National Nature Reserve Pingwu China; 5 State Key Laboratory of Biocontrol and Guangdong Key Laboratory of Plant Resources, School of Life Sciences, Sun Yat-sen University, No. 135, Xin-Gang-Xi Road, Guangzhou 510275, Guangdong, China Sun Yat-sen University Guangzhou China; 6 Center of Conservation Biology, Core Botanical Gardens, South China Botanical Garden, Chinese Academy of Sciences, Guangzhou 510655, Guangdong, China Center of Conservation Biology, Core Botanical Gardens, South China Botanical Garden, Chinese Academy of Sciences Guangzhou China

**Keywords:** Compositae, floral micromorphology, taxonomy, Xuebaoding National Nature Reserve

## Abstract

*Sinoseneciopingwuensis* (Asteraceae, Senecioneae), a new species from Pingwu county in northern Sichuan, China, is described and illustrated. This species is distinguished in *Sinosenecio* by having leathery, glabrous, ovate or ovate-oblong leaves often pinnately-veined and solitary capitula 2.3–4.3 cm in diameter, a unique character combination hitherto never recorded in the genus. Two floral micromorphological characters (configuration of filament collar of stamens and anther endothecial cell wall thickenings) and achene surface features of the new species are reported. Color photographs of living plants and a distribution map are also provided for the new species.

## ﻿Introduction

**Figure 1.**
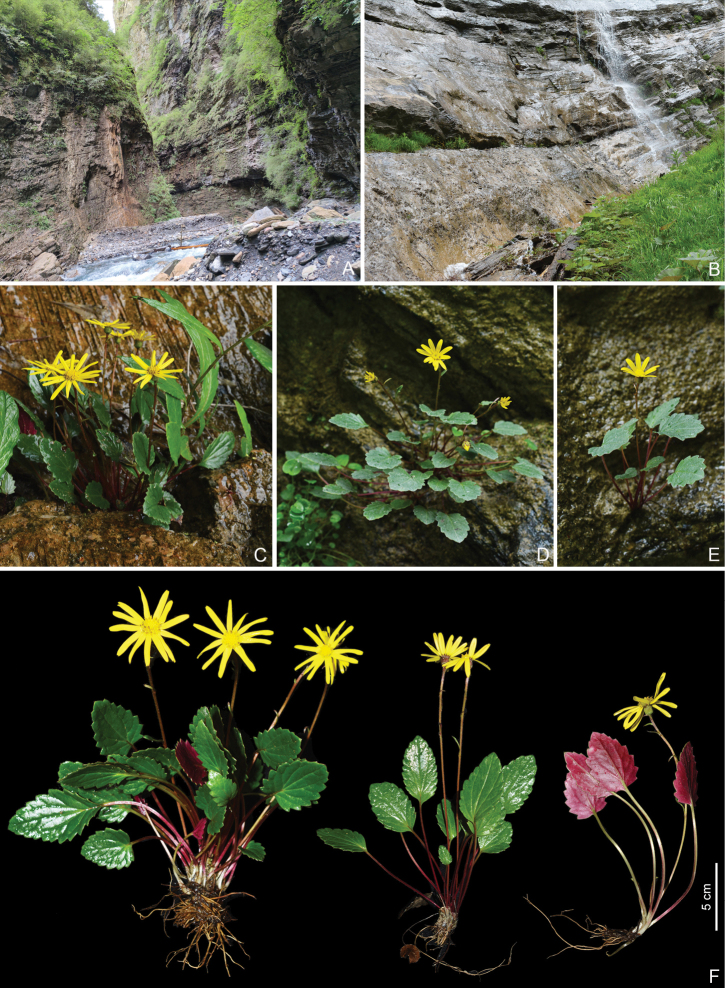
*Sinoseneciopingwuensis* sp. nov. in the wild (China, Sichuan province, Pingwu county, the type locality) **A, B** habitat **C–E** habitat and habit **F** habit. Photographed by W.Q. Fei.

**Figure 2.**
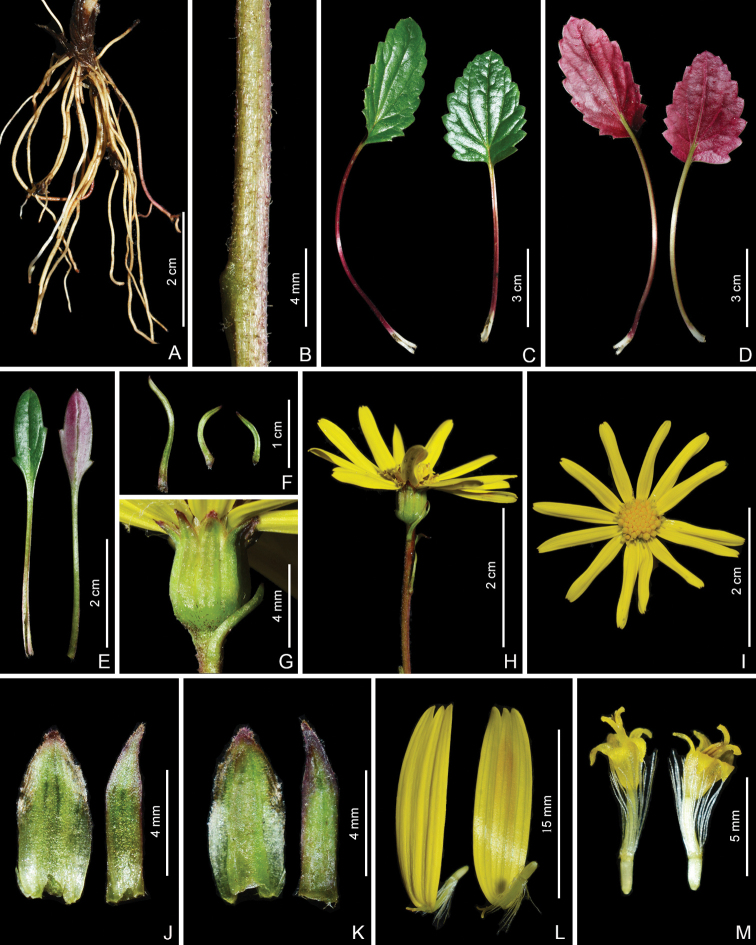
*Sinoseneciopingwuensis* sp. nov. in the wild (China, Sichuan province, Pingwu county, the type locality) **A** roots **B** portion of stem **C** radical leaves (adaxial side) **D** radical leaves (abaxial side) **E** radical leaf (left: adaxial side; right: abaxial side) **F** bracts on the scape **G** close-up of capitulum **H** capitulum (lateral view) and portion of scape **I** capitulum (top view) **J** phyllaries (adaxial side) **K** phyllaries (abaxial side) **L** ray florets **M** disc florets. Photographed by W.Q. Fei.

**Figure 3.**
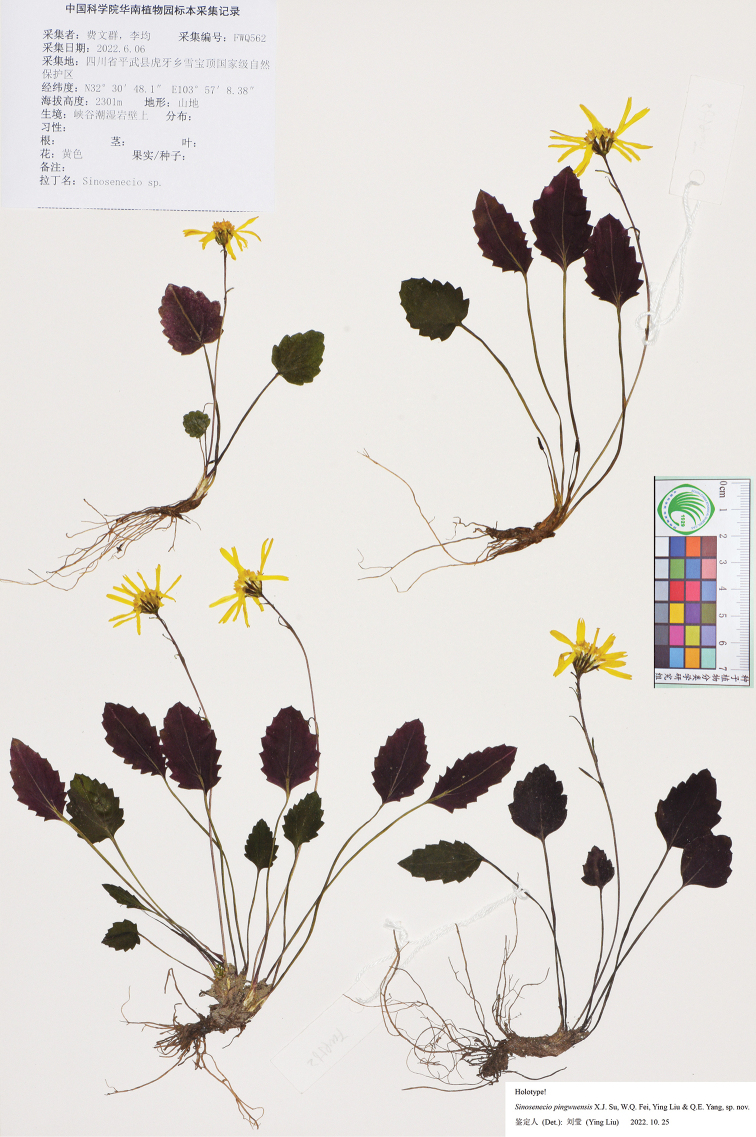
Holotype sheet of *Sinoseneciopingwuensis* sp. nov.

During a botanical trip in 2016 in connection with the biodiversity survey of the Xuebaoding National Nature Reserve in Pingwu county in northern Sichuan, China, we discovered an unusual population of *Sinosenecio* B. Nord. (Asteraceae, Senecioneae) (Figs 1–3). The plants are distinguished in *Sinosenecio* by having leathery, glabrous, ovate or ovate-oblong leaves often pinnately-veined, and solitary capitula 2.3–4.3 cm in diameter, a unique character combination hitherto never recorded in the genus. We therefore determined that the population in question represents a new species, which we describe below.

## ﻿Taxonomic treatment

### ﻿ Sinosenecio pingwuensis

Taxon classificationPlantaeAsteralesAsteraceae

Xiu J.Su, W.Q.Fei, Ying Liu & Q.E.Yang sp. nov.

4DCDA901-014E-5547-B2F3-6D5FA083C4CB

urn:lsid:ipni.org:names:77311810-1

Figs 1 , 2 , 3

#### Type.

China. Sichuan province: Pingwu county, Huya town, Xuebaoding National Nature Reserve, on moist rocky cliffs in valley, alt. ca. 2300 m, 6 June 2022, *W. Q. Fei & J. Li 562* (holotype: IBSC; isotypes: CDBI, PE, SYS). Fig. 3.

#### Diagnosis.

*Sinoseneciopingwuensis* is distinguished in the genus by having leathery, glabrous, ovate or ovate-oblong leaves often pinnately-veined and solitary capitula 2.3–4.3 cm in diameter.

#### Description.

Scapigerous herbs. Rhizomes 2–5 mm in diameter, clad in persistent petiole bases; collar densely sericeous-villous. Stems 1 or 2, erect, scapiform, 11–20 cm tall, simple, purplish, sparsely pubescent, more densely so at base and in upper part below the capitulum, sometimes glabrescent in the middle part. Leaves radical, rosulate, long petiolate; petioles 3.3–10 cm long, basally expanded, sparsely villous or pubescent, densely so at base, often glabrescent in the middle and upper parts; blades ovate or ovate-oblong, rarely broadly ovate, 1–4.5 × 0.9–3 cm, leathery, abaxially purplish, adaxially green or dark green, glabrous on both sides, palmately 5–7-veined or pinnately-veined due to some of the main veins arising from the mid-rib above the base, veins conspicuous adaxially, ± raised abaxially, margin dentate, rarely mucronulate, base truncate, rounded or cuneate, apex acute or obtuse. Capitula terminal, solitary, radiating, 2.3–4.3 cm in diameter; scape often bearing 2–6 sessile, linear bracts 4–16 mm long in the middle and upper parts, rarely the lowest one with petiole 1–3 cm long. Involucres campanulate, 7–9 × 5.5–7.5 mm, ecalyculate; phyllaries 8–13, ovate-oblong to linear-oblong, 1.5–3.5 mm wide, herbaceous, sparsely pubescent with blackish purple hairs in the middle and at base, sometimes glabrescent, margin scarious, apically purplish, ciliate, acuminate. Ray florets 11–13; corolla tube 2–3 mm long, glabrous; lamina yellow, oblong, 14–17 × 2–3 mm, 4–7-veined, apically 3-denticulate. Disc florets 33–55; corolla yellow, ca. 6 mm long, with ca. 3 mm long tube and funnelform campanulate limb; lobes ovate-oblong, ca. 1 mm long, apically acuminate. Anthers oblong, ca. 2 mm long, basally obtuse. Style branches 0.5 mm long, recurved, apically truncate, papillose. Achenes cylindrical, ca. 2.5 mm long (immature), smooth, glabrous, ribbed. Pappus white, 5–6 mm long.

#### Floral micromorphological characters and achene surface features.

**Figure 4.**
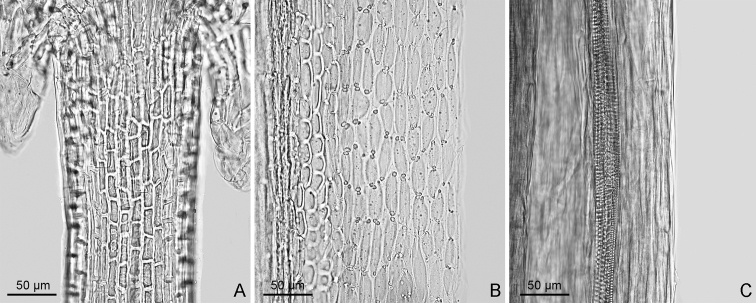
Two floral micromorphological characters (**A, B**) and achene surface feature (**C**) of *Sinoseneciopingwuensis* sp. nov. **A** uniformly-sized cells of filament collar of stamens **B** strictly polar anther endothecial cell wall thickenings **C** smooth achene surface. All from *W.Q. Fei & J. Li 562* (IBSC, SYS) from Pingwu county in northern Sichuan province, China.

The filament collar of stamens in *Sinoseneciopingwuensis* consists of uniformly-sized cells (Fig. 4A) and the anther endothecial cell wall thickenings are strictly polar (Fig. 4B). The achene is glabrous and smooth (Fig. 4C).

#### Phenology.

Flowering in June; fruiting in July.

#### Etymology.

The specific epithet, “*pingwuensis*”, refers to the type locality of the new species, i.e. Pingwu county in northern Sichuan, China.

#### Distribution and habitat.

**Figure 5.**
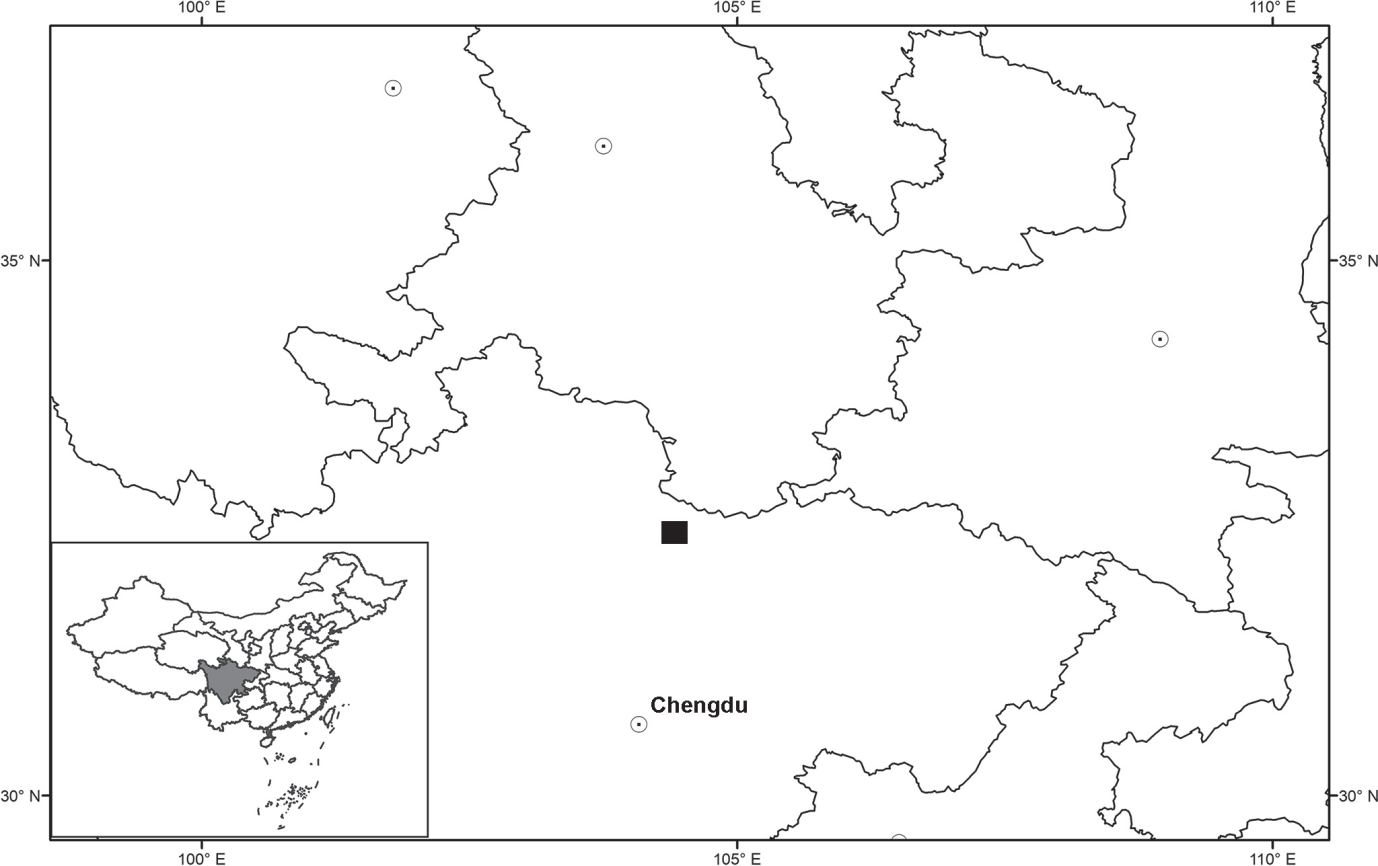
Distribution of *Sinoseneciopingwuensis* sp. nov. (black square).

*Sinoseneciopingwuensis* is currently known only from its type locality, i.e. Pingwu county in northern Sichuan, China (Fig. 5). It grows on moist rocky cliffs along stream sides in a valley at an altitude of ca. 2300 m above sea level.

#### Conservation status.

The currently only known population of *Sinoseneciopingwuensis* at the type locality comprises ca. 80 individuals growing on rocky cliffs. They are scattered within ca. 1 km along a valley. Although the population is located in the Xuebaoding National Nature Reserve, some human activities, road building in particular, may destroy the habitat of the population and, thus, severely affect the survival of this species. According to the IUCN Red List Categories and Criteria (IUCN 2012), the new species should be categorised as Critically Endangered (CR).

#### Notes.

The genus *Sinosenecio*, as defined by Chen et al. (2011), comprises two major species assemblages with different configurations of anther endothecial cell wall thickenings (polar and radial vs. strictly polar), different base chromosome numbers (*x* = 24, rarely 13 vs. *x* = 30) and different geographical distributions (central and southern China vs. areas largely surrounding the Sichuan basin in south-western China) (Liu 2010; Liu and Yang 2011a, b, 2012; Liu et al. 2019; Zou et al. 2020; Chen et al. 2022; Peng et al. 2022). Judging from its strictly polar anther endothecial cell wall thickenings and its occurrence only in Pingwu county at the northern margin of the Sichuan basin, *S.pingwuensis* should belong to the latter assemblage, in which 14 species are currently recognised, including *S.homogyniphyllus* (Cumm.) B. Nord., the type species of *Sinosenecio* (Liu 2010; Chen et al. 2011; Chen et al. 2022). Regrettably, we have been unable to check the chromosome number of *S.pingwuensis* due to our failure in transplanting living plants to obtain actively growing roots for squashing. From its configuration of strictly polar anther endothecial cell wall thickenings, *S.pingwuensis* should have a somatic chromosome number (2*n*), based on *x* = 30, very likely 2*n* = 60, the commonest somatic chromosome number in this assemblage (Liu and Yang 2011a). In *Sinosenecio*, the strictly polar anther endothecial cell wall thickenings correlate well with the base chromosome number of *x* = 30 (Liu 2010; Liu and Yang 2011a, b).

In the same valley where *Sinoseneciopingwuensis* occurs, we discovered another hitherto undescribed species of *Sinosenecio*. This species and *S.pingwuensis* should belong to the same species assemblage of the genus. Both prefer shaded and moist microhabitat and grow on rocky cliffs. Although they do not grow strictly in the same community, some individuals of them are less than 100 m away from each other and they begin to flower at the same time (in June). We did not observe, however, any morphologically putative hybrids between them. This is probably due to isolation via intrinsic post-zygotic barriers. We will report this undescribed species elsewhere.

## Supplementary Material

XML Treatment for Sinosenecio pingwuensis
